# Tumorigenic Effects of Tamoxifen on the Female Genital Tract

**DOI:** 10.4137/cpath.s487

**Published:** 2008-03-01

**Authors:** Kaei Nasu, Noriyuki Takai, Masakazu Nishida, Hisashi Narahara

**Affiliations:** Department of Obstetrics and Gynecology, Faculty of Medicine, Oita University, Oita, Japan

**Keywords:** tamoxifen, tumorigenicity, ovary, uterus, estrogenicity, breast cancer

## Abstract

Tamoxifen is widely used for endocrine treatment and breast cancer prevention. It acts as both an estrogen antagonist in breast tissue and an estrogen agonist in the female lower genital tract. Tamoxifen causes severe gynecologic side effects, such as endometrial cancer. This review focuses on the effects of prolonged tamoxifen treatment on the human female genital tract and considers its tumorigenicity in the gynecologic organs through clinical data analysis. Tamoxifen is associated with an increased incidence of benign endometrial lesions such as polyps and hyperplasia and a two- to four-fold increased risk of endometrial cancer in postmenopausal patients. Moreover, the incidence of functional ovarian cysts is significantly high in premenopausal tamoxifen users. To prevent tamoxifen from having severe side effects in gynecologic organs, frequent gynecological examination should be performed for both premenopausal and postmenopausal patients with breast cancer who are treated with this drug.

## Introduction

**Figure 1.**
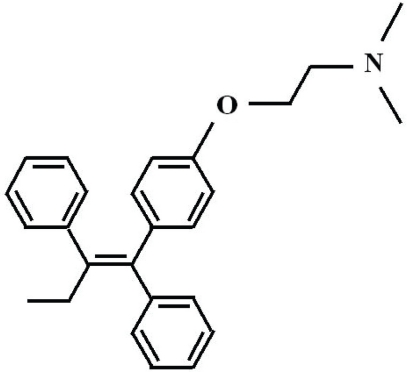
Molecular structure of tamoxifen.

**Figure 2.**
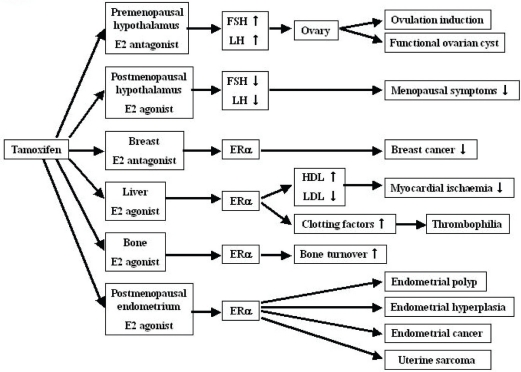
Pleiotropic effects of tamoxifen pointing to organ-specific beneficial or deleterious effects.

Tamoxifen is a nonsteroidal triphenylethyl compound that belongs to a class of selective estrogen receptor modulators (SERMs) (Fig. 1), binds to estrogen receptors (ERs) and elicits estrogen agonist or antagonist responses, depending on the target tissue (Fig. 2) (MacGregor and Jordan, 1998; Pasqualini et al. 1998). Its estrogen antagonist properties have made tamoxifen an important treatment modality for patients with breast cancer, especially those whose tumors are positive for ERs. At present, tamoxifen is one of the most widely prescribed drugs in the world (ACOG Committee Opinion, 2006).

Tamoxifen was approved in 1977 by the US Food and Drug Administration for the treatment of metastatic breast cancer in postmenopausal patients. Tamoxifen was also found to suppress ER-positive breast cancer in postmenopausal women, to increase the disease-free interval, and to reduce the incidence of contralateral breast cancer in breast cancer patients (Early Breast Cancer Trialists’ Collaborative Group, 1992). It is also effective in premenopausal breast cancer patients (Osborne, 1998; Clarke, 2006). Early treatment for breast cancer metastasis has been found to delay disease progression (Nicolini et al. 1997). Currently, tamoxifen is an option along with aromatase inhibitors as the endocrine treatment of choice in all stages of breast cancer in both pre- and postmenopausal women (Osborne, 1998; Clarke, 2006). Fisher and colleagues, (1989) recommended that all low-risk patients with breast cancer receive adjuvant therapy, and a large number of premenopausal and postmenopausal women have been placed on 3- to 5-year regimens of tamoxifen. In addition, prophylactic use of tamoxifen resulted in a 45% reduction in the incidence of ER-positive breast cancer in healthy women, according to the Breast Cancer Prevention Trial of the National Surgical Adjuvant Breast and Bowel Project (Fisher et al. 1998). Therefore, in 1998 the US Food and Drug Administration approved the use of tamoxifen as a chemopreventive option in both premenopausal and postmenopausal women.

Efficacy of tamoxifen in breast cancer is due to its anti-estrogen properties, but it may also exert a weak estrogenic effect resulting in a variety of lesions in the female genital tract. Many ex vivo and in vivo studies have shown quite strong estrogenic-like activity in the endometrium, vagina, liver, and bones (Patterson et al. 1982; Wolf and Jordan, 1992). Tamoxifen blocks ERs in the hypothalamus, leading to the inhibition of estrogen feedback, which leads to increases in the production of gonadotropin-releasing hormone (GnRH), follicle-stimulating hormone (FSH), and luteinizing hormone (LH). Consequently, the ovaries may become hyperstimulated, form cysts, and produce more estrogen. FSH and LH levels fluctuated during the treatment period. Estradiol (E_2_) levels were significantly higher in the study group than in the control group (Sherman et al. 1979). Conversely, tamoxifen manifests estrogen agonist activity in the skeleton and uterus, as well as on a number of intermediate markers of cardiovascular risk.

Tamoxifen has long been considered a safe medication with few serious side effects. However, it has become clear in the past decade that prolonged use of this agent is associated with significant gynecological complications (Table 1). The potential adverse effects of the drug include the development of endometrial cancer (Killackey et al. 1985; Fisher et al. 1994; Clarke et al. 1998; Bernstein et al. 1999), endometrial polyps (Corley et al. 1992; Lahti et al. 1993; Kedar et al. 1994), adenomyomatous polyp (Nasu et al. 1997), adenomyosis (Cohen et al. 1995), leiomyoma (Dilts et al. 1992; Leo et al. 1994; Kang et al. 1996), cervical polyps, and ovarian cysts (Sawka et al. 1986; Cohen et al. 1994a; Barbieri et al. 1993; Shushan et al. 1996a; Terada et al. 1993; Nasu et al. 1999). Of these diseases, tamoxifen-associated endometrial pathologies have been evaluated exclusively. There is sufficient clinical data to suggest that postmenopausal tamoxifen therapy may increase the risk of developing benign and malignant endometrial pathologies (Cohen et al. 1998a). It is interesting that some of these ovarian cysts are functional, such as follicular or luteinized cysts (Dilts et al. 1992; Barbieri et al. 1993; Shushan et al. 1996a; Terada et al. 1993). Tamoxifen was listed in 1996 as a human carcinogen by the International Agency of Research on Cancer (International Agency of Research on Cancer, 1996).

This review focuses on the effects of prolonged tamoxifen treatment on the adult human female genital tract and considers its tumorigenicity in the gynecologic organs based on an analysis of clinical data.

## Action Mechanism of Tamoxifen on Target Organs

Tamoxifen binds to the ER with a Kd of <2 nM, which is ∼20-fold lower than that of 17β-estradiol (Capony and Rochefort, 1978). Administered as a single oral dose of 20 mg, tamoxifen is rapidly absorbed, with its concentration peaking in ∼5 hours. The terminal elimination half-life is ∼5–7 days. Steady-state concentrations in plasma are reached after ∼4 weeks of tamoxifen therapy in women. Tamoxifen is extensively metabolized after oral administration: ∼65% of the administered dose is excreted over 2 weeks, primarily through the feces. Tamoxifen is excreted mainly as polar conjugates, which account for ∼70% of the elimination products. Tamoxifen is hydrated by cytochrome P450 (CYP) 2D6 to the potent metabolites 4-hydroxytamoxifen and 4-hydroxy-N-desmethyl tamoxifen (endoxifen). The major metabolite, 4-hydroxy-N-desmethyl tamoxifen, is similar in biological activity to tamoxifen (Zeneca Pharmaceuticals, 1998). CYP2D6 activity is considered as a determinant of tamoxifen efficacy and adverse effects (Goetz et al. 2005). Breast cancer patients who were poor metabolizers of CYP2D6 had a worse clinical outcome and fewer adverse effects compared with those who were extensive metabolizers of CYP2D6.

The cell-specific effects of tamoxifen in genital tissues and its divergent effects in premenopausal and postmenopausal women are complex, thus making it difficult to determine what defines the biologic effect (i.e. agonist or antagonist) on a specific gynecologic organ or tissue. Tamoxifen also interacts with cellular proteins other than the estrogen receptor, such as protein kinase C, calmodulin, transforming growth factor-β, insulin-like growth factor-I, phosphoinositide kinase, P-glycoprotein, and membrane-associated proteins through the ER-independent pathway (Lam, 1984; O’Brian et al. 1985; Butta et al. 1992; Laatikainen et al. 1995; Cabot et al. 1997; Duk et al. 1997; Elkas et al. 1998; Friedman, 1998; Zhao et al. 1998).

**Figure 3.**
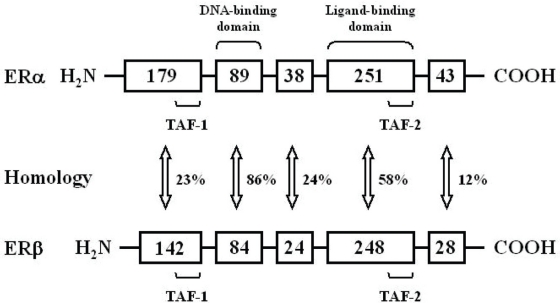
Comparison of the two estrogen receptor molecules. The estrogen receptor consists of six functional domains. Estrogens manifest their biological activity through two distinct receptors, ERα and ERβ. The numbers in the boxes indicate numbers of amino acids. Homology between the distinct domains of the receptors is indicated. It is believed that the binding of different ligands induces structural alterations within the estrogen receptor and that the cells differ in their ability to recognize these conformations. TAF: transcription activating function.

In 1996, a second isoforms, ERβ, was discovered (Kuiper et al. 1996; Mosselman et al. 1996). One possible explanation for the tissue-selective activity of different ER ligands is that they interact with different receptors. In addition, splice variants of each of these receptors have been observed, allowing them to express various isoforms of ERα and ERβ in different tissues (Ogawa et al. 1998). ERβ is expressed in tissues other than ERα, and both ERs have different ligand binding properties. These differences might contribute to the selective action of tamoxifen and other SERMs in different tissues. ERβ transcripts have been detected in tissues such as prostate, ovaries, and lungs, as well as in various parts of the central and peripheral nervous systems. In contrast, ERα is predominantly detected in the pituitary gland, ovaries, uterus, kidneys, adrenals, and mammary glands (Kuiper et al. 1996; Kuiper and Gustafsson, 1997). These differences in the distribution of ERα and ERβ may explain the selectivity of the compounds. Tamoxifen has a similar affinity to both receptors (Kuiper and Gustafsson, 1997). Some scientists believe that uterotrophic activity of tamoxifen is caused by ERβ. Both receptors have identical DNA-binding domains, but within the ligand-binding domain, the amino acid sequence diverges considerably (Kuiper et al. 1996; Kuiper and Gustafsson, 1997) (Fig. 3). Thus, although both receptors have an equivalent affinity to 17β-estradiol with regard to other substances, there are important differences. Tamoxifen acts as an antagonist when both the transactivating domains, transcription activating function (TAF)-1 and TAF-2, are suppressed, but as an agonist when the TAF-1 activation overcomes TAF-2 inhibition.

Cell-specific effect of tamoxifen in the same tissue can be explained by the tripartite theory of Katzenellenbogen et al. (1996), who studied the pharmacologic basis for the cell-and promoter specific action of steroid hormones. They referred to the cell and tissue selectivity that steroid hormones display as a tripartite system comprising ligand, receptor, and effector. Their results showed that molecular elements within the cell nucleus interact with the ligand-receptor complex and influence ER transcriptional response to the ligand. The biocharacter of the ligand (i.e. agonist-antagonist balance) is determined principally through this receptor-effector coupling (O’Brian et al. 1985). This molecular explanation of the agonistic and antagonistic effects of the ligand-binded ER is further supported by others (Brzozowski et al. 1997; Parker, 1998). Hormone binding to the ligand-binding domain of the ER initiated a series of molecular events culminating in the activation or repression of target genes. Each ligand induces a distinct conformation in the transactivation domain, creating an interacting surface to which coactivators are likely to bind. Several candidate coactivating proteins have been identified, including receptor-interacting proteins RIP-140 and RIP-160, L7SPA, and steroid receptor coactivator-1 (SRC-1) (Onate et al. 1995; Horwitz et al. 1996; Jackson et al. 1997; Shah and Rowan, 2005). These proteins interact with receptors only in the presence of their respective ligands, providing structural evidence for the mechanism of repression or activation of target genes (Parker, 1998).

## Ovary

In breast cancer patients, ovarian cyst formation during prolonged tamoxifen treatment (Cohen et al. 1994a; Barbieri et al. 1993; Kedar et al. 1994; Shulman et al. 1994; Nasu et al. 1999) and in series of tamoxifen-treated breast cancer patients (Cohen et al. 1996; Shushan et al. 1996a) has been reported. Ovarian cysts also have been described in a breast cancer prevention study (Powles et al. 1994). These reports described a heterogeneous group of ovarian pathologies with numerous histologic diagnoses, but they did not assess hormones or define menopausal status. In premenopausal patients, tamoxifen disrupts the menstrual cycle and causes functional ovarian cysts (Cohen et al. 1994a; Hochner-Celnikier et al. 1995). The ovarian pathology in these instances includes simple cysts, follicular cysts, luteinized follicular cysts, and corpus luteum cysts (Cohen et al. 1994a; Hochner-Celnikier et al. 1995; Shushan et al. 1996a). Some studies suggest that benign ovarian pathologies may be expected in most premenopausal tamoxifen users (Cohen et al. 1994a). It is interesting that these cysts regress if tamoxifen is withdrawn (Shushan et al. 1996a) or if premenopausal patients are treated with GnRH agonists during tamoxifen treatment (Cohen et al. 1994a; Shushan et al. 1996b). These lesions, although benign, may be complicated by torsion or cystic necrosis and may pose a diagnostic dilemma in patients at risk of ovarian metastases from breast cancer or of primary ovarian cancer (Cohen et al. 1994a).

In premenopausal women, these cysts may be associated with hyperestrogenism (Cohen et al. 1994a; Hochner-Celnikier et al. 1995). In the ovaries of premenopausal patients, tamoxifen stimulates estrogen production by affecting the hypothalamic-pituitary-ovarian feedback mechanism (Kedar et al. 1994). The hyperestrogenemia described during tamoxifen therapy may reflect a simultaneous maturation of multiple ovarian follicles or an enhanced gonadotropin stimulation of a single maturing follicle (Sherman et al. 1979). Such a phenomenon may lead to an increased risk of fibroid ovaries and ovarian cysts (Cohen et al. 1994a). However, the mechanism by which tamoxifen stimulates the development of ovarian cysts has not yet been fully explored. It was suggested that the mechanism by which tamoxifen induces ovarian cysts in premenopausal women could be by a direct action on the ovaries to stimulate excessive growth of ovarian follicles, resulting in elevated estradiol levels (up to 3,700 pg/ml), throughout all phases of the menstrual cycle (Terada et al. 1993). Mourits et al. (1999) performed a prospective study using transvaginal ultrasound with hormonal assessment and reported ovarian cysts in 40% of premenopausal women during tamoxifen treatment, whereas none of the postmenopausal patients developed cystic ovaries. In patients with regular menstrual cycles during tamoxifen treatment, 81% developed ovarian cysts. In these premenopausal women with cystic ovaries, the serum estrogen levels were markedly elevated, with gonadotropin concentrations either unchanged or slightly increased.

Sawka et al. (1986) reported that 7 of 84 premenopausal women being treated with tamoxifen for breast cancer developed cystic enlargement of the ovaries. It is uncertain how many of these cases will require surgical intervention. Shushan et al. (1996a) reported that 5 of 79 tamoxifen treated premenopausal women with breast cancer had cystic enlargement of the ovaries. They also reported that, in 8 of 11 patients, the ovarian cystic enlargement disappeared after the cessation of tamoxifen treatment. Barbieri et al. (1993) reported a case of a 45-year-old woman with breast cancer treated with tamoxifen for 2 years. She had bilateral functional ovarian cysts and torsion of unilateral adnexa, and underwent surgical treatment. Terada et al. (1993) also reported a case of a large ovarian follicular cyst with torsion, whose serum estradiol level was significantly increased. Sadan et al. (2001) reported that 7 of 10 (70%) women with tamoxifen administration developed ultrasonographically benign ovarian cysts ranging from 1.5 to 6.0 cm in diameter. One woman underwent surgery to remove an enlarging cyst. In all of the other patients, ovarian cysts disappeared within three months after the cessation of therapy. We have also reported the torsion of an ovarian functional cyst in a premenopausal breast cancer patient who was treated surgically (Nasu et al. 1999).

Although torsion of a cystic ovary during tamoxifen treatment has been described (Barbieri et al. 1993; Nasu et al. 1999), surgical intervention is rarely required, and functional asymptomatic monolocular cysts in these patients should be followed conservatively (Mourits et al. 2001). The discontinuation of tamoxifen usually leads to the gradual reduction and disappearance of these lesions (Cohen et al. 1994a).

Whereas tamoxifen in postmenopausal patients induces ovarian cystic tumors and endometriomas (Kedar et al. 1994; Shushan et al. 1996a; Varras et al. 2003), Cohen et al. (1996) reported that 10 of 16 tamoxifen-treated women who had undergone hysterectomy had ovarian neoplasms. Four of these women had serous cystadenomas, and two had serous cystadenofibromas. Other ovarian tumors in this group included an endometrioid adenocarcinoma (Cohen et al. 1994b), a Brenner tumor, a thecoma, and ovarian fibromas. It is controversial whether or not tamoxifen users risk developing ovarian cancer (Cohen et al. 1996; Ismail, 1999; Lewis 2000).

## Uterine Corpus

### Mechanism of endometrial tumorigenesis

The possible mechanisms involved in the endometrial carcinogenicity of tamoxifen are quite complex. The molecular machinery involved in the induction of endometrial polyps, hyperplasia, and endometrial carcinoma by tamoxifen may differ from that of estrogens (Webb et al. 1995). Regarding the action of tamoxifen in the endometrium, we propose this mechanism: tamoxifen binds to the ER in the estrogen-binding domain, located in the functional domain E of the ER, and forms dimers of activated ERs (receptor dimerization). These dimers bind to the specialized ER response elements (EREs) of the DNA in the endometrial cells. This binding allows the activation of the ER region TAF-1 function, which mediates the activation of the estrogen-independent transcriptional activating function (Fig. 3). It has been reported that tamoxifen and its metabolites are concentrated in the endometrium, especially with hyperplastic changes (Giorda et al. 2000). The estrogenic activity of tamoxifen is cell type-specific in that it stimulates cell growth and the transcription of a number of estrogen-regulated genes in the uterus but not in the breast (Ramkumar and Adler, 1995; Webb et al. 1995). The mitogen function of tamoxifen in the endometrium is perhaps explained by the activation of proto-oncogenes, which occurs exclusively by the region TAF-1 function. In contrast, in the breast the expression of proto-oncogenes depends on the region TAF-2 function, which is activated only by estrogens. The overexpression of proto-oncogenes caused by tamoxifen may be responsible for carcinogenesis in the endometrium. It has been reported that tamoxifen induces endometrial K-ras oncogene codon 12 mutations, which are considered important in endometrial carcinogenesis (Sasaki et al. 1993; Hachisuga et al. 2005; Wallen et al. 2005). Tamoxifen also modulates the expression of a variety of endometrial genes, including tumor protein p53, nuclear factor-κB transcription factor subunit p65 (RelA), myelocytomatosis viral oncogene homologue (myc), epidermal growth factor receptor, and β-catenin (Gielen et al. 2005).

Tamoxifen is metabolized in the human liver to α-OH-tamoxifen, N-des-tamoxifen, tamoxifen N-oxide, and 4-hydroxytamoxifen (Randerath et al. 1994; Jarman et al. 1995). Among these tamoxifen metabolites, it is reported that α-OH-tamoxifen is sulfonated by hydroxysteroid sulfotransferases (Shibutani et al. 1998a; Shibutani et al. 1998b) and reacts with the exocyclic amino group of guanine in DNA, resulting in the formation of two trans and two cis diastereoisomers of α-(N^2^-deoxyguanoxinyl)tamoxifen (dG-N^2^-tamoxifen), which have been found in patients treated with tamoxifen (Shibutani et al. 1999). dG-N^2^-tamoxifen adducts display a high miscoding and mutagenic potential and generate primarily G- to T-transversions in mammalian cells (Shibutani and Dasaradhi, 1997; Terashima et al. 1999). Such tamoxifen adducts, if not repaired (Shibutani et al. 2000b), may cause mutations, leading to the development of endometrial cancers. Significant levels of dG-N^2^-tamoxifen adducts have been detected in the endometria of certain women treated with tamoxifen (Shibutani et al. 1999; Shibutani et al. 2000a). The biotransformation of tamoxifen into α-hydroxytamoxifen, 4-hydroxytamoxifen, and N-desmethyltamoxifen has been demonstrated in an endometrial explant culture (Sharma et al. 2003). Kim et al. (2005) reported that the addition of α-hydroxytamoxifen to the endometrial explant culture induced tamoxifen-DNA adduct. In a study using the accelerator mass spectrometry, tamoxifen bound irreversibly to DNA, forming DNA adducts at low levels in the endometrium (Martin et al. 2003).

Clinical data indicate that tamoxifen therapy may cause an increased risk of endometrial pathology in postmenopausal women but not in premenopausal women. It has been demonstrated that tamoxifen exerts a proapoptotic effect on cultured endometrial cells in the presence of estrogen (Stackievicz et al. 2001). On the other hand, tamoxifen shows a weak anti-apoptotic effect on these cells in the absence of estrogen. In 2007, Singh et al. (2007) proposed a dual mechanism of action highlighted by the different patterns of endometrial carcinoma sub-types. Tamoxifen may initially be pro-estrogenic in the endometrium giving rise to elevated type-1 endometrioid adenocarcinoma, whereas after long-term use, there is an increase of type-2 disease or malignant mixed Müllerian tumors (MMMTs) associated with a hormone-independent mechanism of action.

### Tamoxifen-induced endometrial pathology

Various histopathological findings ranging from proliferative endometrium, hyperplasia, and polyps to carcinoma have been found in tamoxifen-treated patients. Combining the results of four prospective studies, Assikis and Jordan, (1995) reported polyps and hyperplasia in 16% of women on tamoxifen. Juneja et al. (2002) found polyps and hyperplasia in 20% of women on tamoxifen. It has been reported that two-thirds of the tamoxifen-treated patients have no pathologic endometrial changes and one-third have tamoxifen-associated endometrial changes including endometrial polyps, hyperplasia, metaplasia, and cancer (Deligdisch et al. 2000). It has been reported that 10% of tamoxifen-treated patients will develop tamoxifen-induced endometrial pathology within 5 years, leading to operative intention (Gerber et al. 2000).

In premenopausal women there is nearly no excess risk of adverse endometrial effects (Cheng et al. 1997; Fisher et al. 1998). Effect of tamoxifen on the endometrium varies with the ambient E_2_ concentration in that it will function as an estrogen agonist only in postmenopausal women and thus may cause endometrial pathologies (Gal et al. 1991; Lahti et al. 1993; Lahti et al. 1994; Cohen et al. 1994a; Kedar et al. 1994). The proliferative activity in benign endometrial epithelium is higher in tamoxifen users than in non-users (Elkas et al. 2000; Mourits et al. 2002), suggesting that this phenomenon may play a role in the higher incidence of endometrial cancer in postmenopausal tamoxifen users. Proliferative effect of tamoxifen on the endometrium has been supported by molecular data. The expression of both ER and PR was found to be consistently positive in endometria from women treated with tamoxifen (Cano and Hermenegildo, 2000). This positivity was reported to be even higher than that found in a control group of premenopausal women (Kommoss et al. 1998). Tamoxifen also mimicked estradiol treatment in upregulating ER, c-fos, and glyceraldehyde phosphate dehydogenase mRNAs, together with other estrogen-induced genes (Rivera-Gonzalez et al. 1998; Robertson et al. 1998). The bromo-deoxyuridine index, an indicator of cell mitogenesis, was shown to be increased in endometrial cells from tamoxifen-treated uteri (Carthew et al. 1999). In this connection, the expression of proliferation markers, e.g. Ki67, was potentiated by tamoxifen in human endometrium (Elkas et al. 1998). An increased susceptibility to genetic lesions associated with carcinogenesis linked to tamoxifen was suggested by a study on endometrium of surgically post-menopausal cynomolgus macaques, where the drug induced p53 positivity, although at a lower level than conjugated estrogen (Isaksson et al. 1999).

### Endometrial hyperplasia

An increased incidence of endometrial hyperplasia up to 50% has also been detected by some investigators in samples obtained from tamoxifen-treated breast cancer patients compared with breast cancer patients not being treated with tamoxifen (Gal et al. 1991; Lahti et al. 1993). In a cohort of 61 normal postmenopausal women recruited from the Pilot Breast Cancer Prevention Trial (Kedar et al. 1994), atypical hyperplasia was detected in 16% of the tamoxifen-treated women.

Neven et al. (1989) found proliferative/hyperplastic features in the endometrium of 43% of tamoxifen-treated breast cancer patients compared with 14% of untreated patients. Cohen et al. (1993) found proliferative activity in 19 of 22 endometrial biopsies obtained from a series of 77 asymptomatic tamoxifen treated postmenopausal breast cancer patients. In a study of 16 breast cancer patients receiving tamoxifen, 7 women had mild proliferative endometrium after 16 months of treatment (Neven et al. 1990). The same group ultimately reported that 5 out of 57 women receiving tamoxifen developed clear endometrial hyperplasia or cancer; the endometrium remained atrophic in 24 women (Neven et al. 1998).

On macroscopic examination, tamoxifen-stimulated endometrial hyperplasia is characterized by diffuse endometrial thickening with a Swiss-cheese-like cut surface owing to the presence of multiple intraendometrial cysts of varying size (Ismail, 1994). Microscopic examination confirms that there is diffuse endometrial thickening with cystically dilated glands, which are clearly of endometrial origin and located within the endometrium (Ismail, 1994) rather than beneath it, as has been suggested on the basis of ultrasonographic findings (Goldstein, 1994). Some endometrial glands show glandular budding and other architectural abnormalities. Focally, the glandular epithelium shows mitotic activity, and often also shows mucinous, clear cell, oxyphilic, and other epithelial metaplasias. The endometrial stroma is characteristically fibrotic (Ismail, 1994; Ismail, 1996) with collagen bundles separating stromal cells (Ismail, 1996). Occasional stromal mitoses may also be seen. These microscopic appearances, which indicate an increase in the amount of endometrial tissue and a low but definite proliferative activity, are best interpreted as hyperplasia of the endometrium (Ismail, 1998). Zhao et al. (1998) have shown that endometria of women receiving tamoxifen express adrenomedullin, a growth factor for endothelial cells; they postulated that induction of this angiogenic factor is part of the mechanism by which tamoxifen results in endometrial hyperplasia.

If the hyperplasia is atypical or complex, discontinuation of the drug is advisable. If tamoxifen has to be continued, hysterectomy may be an option.

### Endometrial polyp

Many studies have shown a high frequency of endometrial polyps in tamoxifen-treated postmenopausal breast cancer patients (Neven et al. 1989; De Muylder et al. 1991; Corley et al. 1992; Cohen et al. 1993; Lahti et al. 1993; Ismail, 1994; Kedar et al. 1994; Silva et al. 1994; Cheng et al. 1997; Cohen et al. 1999), but a low frequency of endometrial polyps in tamoxifen-treated premenopausal patients (Hachisuga et al. 1999). Evidence of polyp formation is reported to occur in 8 to 50% of women on tamoxifen (Neven et al. 1989; Gal et al. 1991; Lahti et al. 1993; Ismail, 1994; Kedar et al. 1994), which exceeds the <4% observed in patients with breast cancer not receiving tamoxifen. Neven et al. (1989) reported a sevenfold increase in endometrial polyps among tamoxifen-treated asymptomatic breast cancer patients when compared with patients not treated with tamoxifen. A larger study by Lahti et al. (1993) found endometrial polyps in 36% of treated and 10% of untreated asymptomatic postmenopausal breast cancer patients. Although usually benign, such polyps differ histologically from non-tamoxifen-exposed endometrial polyps.

Cohen et al. (2001) demonstrated that the risk factors for the development of endometrial polyps associated with tamoxifen therapy are older age at menopause, longer duration of breast cancer disease, heavier weight, and thicker endometrium. Especially, the long-tem tamoxifen therapy (>48 consecutive months) has been shown to increase the frequency of endometrial lesion, especially of endometrial polyps (Cohen et al. 1996).

Endometrial polyps that develop in tamoxifen-treated women have unusual macroscopic and microscopic features (Nuevo et al. 1989; Corley et al. 1992; Ismail, 1994). Tamoxifen associated endometrial polyps tend to develop on a background of endometrial hyperplasia (Ismail, 1994). The polyps are often multiple and usually much larger than sporadic polyps (Nuovo et al. 1989; Corley et al. 1992; Ismail, 1994). They may undergo ulceration and infarction, and their consequent irregular friable appearance may mimic malignancy. Gross myxoid change is occasionally seen (Ismail, 1999). On microscopic examination, the polyps comprise abundant fibromyxoid stroma and architecturally abnormal endometrial glands lined mostly by benign epithelium (Ismail, 1994; Neven, 1995; Deligdisch et al. 2000). Proliferative activity is commonly seen in both epithelial and stromal cells. The epithelial cells lining the glands show a range of metaplasias including apocrine, squamoid, mucinous, clear cell, and oxyphil cell metaplasia (Ismail, 1999). There is patchy condensation of the stromal cells around the glands. It has been reported that Bcl-2 and Ki-67 expression was higher in tamoxifen-associated polyps than in postmenopausal untreated polyps (Altaner et al. 2006). These findings suggest that tamoxifen-associated endometrial polyps have higher rates of cell mitotic activity and apoptosis inhibition; these higher rates may play a crucial role in the malignant transformation of such endometrial polyps.

A high prevalence of endometrial carcinoma arises from tamoxifen associated endometrial polyps (Cohen, 2004). From 3 to 10.7% of endometrial polyps resected from tamoxifen-treated postmenopausal patients with breast cancer were found to experience malignant changes (Cohen et al. 1999). In contrast, only 4 endometrial carcinoma arising from endometrial polyps were found in a large study of 1,100 endometrial polyps carried out before the introduction of tamoxifen (Peterson and Novak, 1956). Silva et al. (1994) showed that 77% of postmenopausal patients who developed endometrial carcinoma after tamoxifen treatment had endometrial polyps, whereas 34% patients in a comparable group who did not receive tamoxifen had endometrial polyps. Deligdisch et al. (2000) reported that 15 of 33 endometrial carcinomas that developed in postmenopausal breast cancer patients subsequent to adjuvant tamoxifen treatment were found in endometrial polyps. Nishimura et al. (2001) reported that 5 of 8 endometrial carcinomas in postmenopausal breast cancer patients were associated with polypoid lesions composed of cystically dilated atrophic glands and fibrotic stroma and that no such polyps were found in 2 premenopausal patients. These findings suggest that the polyp-carcinoma sequence plays a partial role in the development of endometrial carcinoma in tamoxifen-treated postmenopausal breast cancer patients (Ismail, 1994).

Adenomyomatous polyp (polypoid adenomyoma) of the endometrium is a rare polypoid lesion in which the stromal component is largely or extensively composed of smooth muscle (Peterson and Novak, 1956). A few cases of adenomyomatous polyps in a postmenopausal patient treated with tamoxifen have been reported (Cohen et al. 1995; Nasu et al. 1997; Takeuchi et al. 2005).

### Endometrial cancer

Endometrial cancer is the most serious uterine condition associated with tamoxifen use. Killackey et al. (1985) reported the first case of endometrial carcinoma in a breast cancer patient who was treated with tamoxifen. After that, a series of case reports suggested an association between tamoxifen and endometrial neoplasia (Jordan, 1998). The first prospective study was published by Neven et al. (1990). Of 16 postmenopausal women treated with tamoxifen for 3 years and followed thereafter by hysteroscopy, 50% maintained inactive atrophic endometrium, while 44% developed proliferation, including 25% polyps and 6% adenocarcinoma. It remains unclear as to whether tamoxifen, because of its estrogen agonistic properties, induces the formation of endometrial cancer or accelerates the growth of pre-existing endometrial cancer. A detection bias must also to be taken into account: gynecological symptoms induced by tamoxifen (vaginal discharge, abnormal bleeding) and/or tamoxifen-related ultrasound phenomena (endometrial thickening) often lead to hysteroscopy, endometrial sampling and/or dilatation and curettage, which might detect latent endometrial cancer that would not have been found if the women had not shown these tamoxifen-induced gynecological symptoms (Baum, 1998).

The National Surgical Adjuvant Breast and Bowel Project (NSABP) prevention trial (P-1) data suggest that the risk ratio for developing endometrial cancer was 2.53 times greater in women using tamoxifen than in women receiving a placebo (Fisher et al. 1998). In addition, the ability of tamoxifen to induce endometrial malignancy as well as other histopathologic conditions appears to differ between premenopausal and postmenopausal women. In the prevention trial of high-risk women, there was no statistically significant difference in endometrial cancer rates between women treated with tamoxifen and those in the placebo group in women aged 49 years or younger; however, in women aged 50 or older, the risk ratio was 4.01 for those treated with tamoxifen versus those receiving placebo. The annual rate was 3.05 malignancies per 1,000 women treated with tamoxifen versus 0.76 malignancies per 1,000 women receiving placebo (Fisher et al. 1998). Another study of women with breast cancer found that premenopausal women, treated or untreated, had no differences in endometrial thickness on ultrasound examination, uterine volume, or histopathologic findings, whereas postmenopausal women treated with tamoxifen had significantly more abnormalities than those who were not treated (Cheng et al. 1997).

The risk of developing endometrial cancer in asymptomatic women is estimated at 1.7 per 1,000 women-years (Koss, 1980). Most studies have found that the increased relative risk of developing endometrial cancer for women taking tamoxifen is two to four times higher than that of an age-matched population (Lahti et al. 1993; Barakat et al. 1994; Cohen et al. 1994a; Fisher et al. 1994; Rutqvist et al. 1995; Curtis et al. 1996; Early Breast Cancer Trialists’ Collaborative Group, 1998). The overall incidence of endometrial cancer in tamoxifen-treated women has been quoted to be 2–3/1,000 (Assikis and Jordan, 1995). The relative risk as calculated by three retrospective randomized clinical trials was 4.1 compared with controls. In one study of the NSABP, the rate of endometrial cancer occurrence among tamoxifen users who were administered 20 mg/day was 1.6 per 1,000 patient years, compared with 0.2 per 1,000 patient years among control patients taking a placebo (Fisher et al. 1994). In a more recent update of NSABP trials of patients with breast cancer, the rate of endometrial cancer was 1.26 per 1,000 patient years in women treated with tamoxifen versus 0.58 per 1,000 in the placebo group (Wickerham et al. 2002). The Stockholm breast cancer study group found a nearly six-fold increase in endometrial carcinomas in patients treated with 40 mg/day tamoxifen for 2–5 years as compared with a control group (Rutqvist et al. 1995). The increased incidence of endometrial cancers was also observed in tamoxifen chemoprevention trials (Fisher et al. 1994; Fisher et al. 1998). It is now considered that the risk level for endometrial cancer in women treated with tamoxifen is dose- and time-dependent. Studies suggest that the stage, grade, histology, and biology of tumors that develop in individuals treated with tamoxifen (20 mg/day) are no different from those that arise in the general population (Fisher et al. 1994; Barakat et al. 1994). However, some reports have indicated that women treated with a higher dosage of tamoxifen (40 mg/day) are more prone to develop more biologically aggressive tumors (Magriples et al. 1993).

Poor histologic phenotypes, including high-grade serous, clear cell, mucinous, or aggressive endometrioid adenocarcinomas, have been reported to be associated with tamoxifen use (Silva et al. 1994; Deligdisch et al. 2000; McCluggage et al. 2003). Magriples et al. (1993) reported that 67% of the corpus cancers that developed in 15 tamoxifen-treated breast cancer patients were high-grade or unfavorable histologic subtypes, compared with 24% of those that developed in 38 breast cancer patients who had not received tamoxifen. A large French case-control study analyzed 135 cases of endometrial cancer diagnosed after breast cancer and 467 matched controls (Mignotte et al. 1998). Breast cancer patients who developed endometrial cancer and had received tamoxifen had a more advanced disease and a poorer prognosis than those with endometrial cancer without prior tamoxifen treatment. Some authors confirmed these findings (Silva et al. 1994; Deligdisch et al. 2000), but others did not (Barakat et al. 1994; Fisher et al. 1994; Rutqvist et al. 1995; Nishimura et al. 2001). Barakat et al. (1994) reported no differences in the stage, grade, or histologic subtype of endometrial carcinomas that develop in breast cancer patients. The NSABP B-14 showed that endometrial carcinomas occurring after tamoxifen therapy do not appear to be of a different type or have a worse prognosis than such tumors in non-tamoxifen-treated patients (Fisher et al. 1994). If the effect of tamoxifen on the endometrium is that of an estrogen agonist, associated endometrial carcinomas could be expected to have prognostic characteristics similar to those associated with unopposed estrogen use. However, the concomitant use of progesterone does not seem to prevent or reverse tamoxifen -associated endometrial changes (De Muylder et al. 1991).

It has been known that tamoxifen can stimulate the proliferation of certain endometrial cancer cell lines (Homesley, 1996). Immunohistochemical studies of the Ki67 index indicate that tamoxifen exerts an antiestrogenic effect on the endometrium in the presence of endogenous estrogen secretion and an estrogenic effect in the absence of estrogen secretion (Hachisuga et al. 1999). This mechanism is further supported by Cross et al. (1990), who described the occurrence of complex atypical hyperplasia in an ovariectomized premenopausal breast cancer patient undergoing tamoxifen treatment. A recent study provides further documentation that the uterotrophic effect of tamoxifen on postmenopausal endometrium may be attributable to overexpression of both ERs and progesterone receptors (Elkas et al. 2000). Oncogene changes, such as K-ras mutation, c-erbB2/neu gene amplification, and cyclin D1 and p53 overexpression have also been observed in tamoxifen-related endometrial cancers (Esteller et al. 1997; Esteller et al. 1999; Boyd, 1996).

In addition to the fact that women with breast cancer are more likely to develop endometrial carcinoma, regardless of tamoxifen treatment, many reports published in recent years have demonstrated a significant association between longer duration of tamoxifen treatment and the appearance of endometrial cancer (Fisher et al. 1994; Ismail, 1994; Jordan and Assikis, 1995; Rutqvist et al. 1995; Curtis et al. 1996; Fisher et al. 1998; Deligdisch et al. 2000). It is now generally accepted that stage, grade, histological type, and other prognostic factors of endometrial cancers associated with tamoxifen (20 mg/day) are not different from those of endometrial cancers found in the normal population tamoxifen (ACOG Committee Opinion, 1996; Homesley, 1996; Love et al. 1999). However, tamoxifen time- and dose-dependently increases the relative risk for endometrial cancer (ACOG Committee Opinion, 1996; MacGregor and Jordan, 1998; Love et al. 1999). The long genesis of cancer in humans makes it likely that short courses of tamoxifen may condition the build-up of cancers that become clinically detectable later. It has been reported that the prognosis of endometrial cancer was significantly worse for long-term tamoxifen users than for non-users, which seems to be due to higher stage and less-favorable histology, such as MMMTs or sarcomas, p53-positive tumors, and ER-negative tumors (Bergman et al. 2000).

Recent studies suggest that low- and high-risk groups of postmenopausal patients may be identifiable before the initiation of tamoxifen therapy for breast cancer (Berliere et al. 2000; Vosse et al. 2002). Pretreatment screening identified 85 asymptomatic patients with benign polyps in 510 postmenopausal patients with newly diagnosed breast cancer (16.7%). All polyps were removed. At the time of polypectomy, two patients had atypical hyperplasias and subsequently underwent hysterectomies. The rest were treated with tamoxifen, 20 mg/day, for up to 5 years. The incidence of atypical hyperplasia was 11.7% in the group with initial lesions versus 0.7% in the group without lesions (p < 0.0001), an 18-fold increase in risk. In addition, polyps developed in 17.6% of the group with initial lesions versus 12.9% in the group without.

In contrast to postmenopausal women, premenopausal women treated with tamoxifen have no known increased risk of uterine cancer (ACOG Committee Opinion, 2000). Stackievicz et al. (2001) suggested that tamoxifen treatment activates apoptotic pathways in the endometrium of premenopausal women, and that the lack of a carcinogenic effect in hormonally active endometrium may be attributable to an active anti-cancerous process and not to menstrual shedding alone.

Uterine sarcomas consisting of mixed Müllerian tumors, leiomyosarcomas, and stromal cell sarcomas are a rare form of uterine malignancy occurring in 2%–5% of all patients with uterine malignancies (Averette and Nguyen, 1995). The mixed Müllerian tumors consist of a glandular component that can be either benign (Müllerian adenosarcoma) or malignant (carcinosarcoma or MMMT), intermixed with nonepithelial elements that are either benign (carcinofibroma) or malignant (carcinosarcoma or MMMT). The mesenchymal component, whether benign or malignant, may exhibit homologous or heterologous differentiation (rhabdo-, lipo-, chondro-, or osteo-differentiations). As both stromal cells and epithelial cells in the uterus express the ER (Vollmer et al. 1990), one might expect tamoxifen to increase the proliferation of both elements, leading to epithelial, mesenchymal, or mixed epithelial-nonepithelial tumors.

In a review of all NSABP breast cancer treatment trials, the rate of sarcoma in women treated with tamoxifen was 17 per 100,000 patient-years versus none in the placebo group (Wickerham et al. 2002). Similarly, in a separate trial of high-risk women without breast cancer taking tamoxifen as part of a breast cancer prevention trial with a median follow-up of 6.9 years, there were 4 sarcomas (17 per 100,000 patient years) in the tamoxifen group versus none in the placebo group (Wickerham et al. 2002). This is compared with the incidence of 1 to 2 per 100,000 patient years in the general population (Mouridsen et al. 1978). Many reports published in recent years have demonstrated a significant association between longer duration of tamoxifen treatment and the appearance of uterine sarcoma (Bergman et al. 2000; Lasset et al. 2001).

Most of the tamoxifen-associated sarcomas reported in the literature were MMMTs (Altaras et al. 1993; Lahti et al. 1993; Cohen et al. 1994a; ACOG Committee Opinion, 1996; Clement et al. 1996; Curtis et al. 1996; Mourits et al. 1998; Deligdisch et al. 2000). Three cases of carcinosarcoma in tamoxifen users were also reported (Treilleux et al. 1999).

Eight cases of uterine adenosarcomas were associated with tamoxifen therapy (Clement et al. 1996; Mourits et al. 1998; Carvalho et al. 2000). Considering the rarity of this disease, it seems that the association of tamoxifen therapy with mesenchymal neoplasm is higher than expected.

Adenofibroma of the endometrium is an uncommon variant of mixed mesodermal tumors, in which the epithelial and stromal components are benign. It most commonly occurs in postmenopausal women (Zaloudek and Norris, 1981). Five cases of uterine adenofibroma in a postmenopausal patient receiving tamoxifen were reported (Huang et al. 1996; Oshima et al. 2002; Hayasaka et al. 2006).

Endometrial stromal sarcoma is a rare uterine mesenchymal tumor reported to account for 0.2% of all uterine malignancies (Koss et al. 1965). Several cases of tamoxifen-associated endometrial stromal sarcoma in postmenopausal breast cancer patients have been reported in the literature (Fisher et al. 1994; Eddy and Mazur, 1997; Mignotte et al. 1998; Wickerham et al. 2002; Saga et al. 2003; Sesti et al. 2005; Hayasaka et al. 2006). These tumors include high-grade endometrial stromal sarcoma (Saga et al. 2003; Sesti et al. 2005), as well as low-grade endometrial stromal sarcoma (Eddy and Mazur, 1997; Mignotte et al. 1998; Wickerham et al. 2002).

### Leiomyoma

Tamoxifen has an estrogenic effect on the myometrium. Several studies have explained the increase in myometrial volume, and the growth of uterine leiomyomas in postmenopausal patients following the administration of tamoxifen (Dilts et al. 1992; Kedar et al. 1994; Cohen et al. 1994a; Leo et al. 1994; Kang et al. 1996). The myomas grew rapidly after the initiation of tamoxifen treatment in the reported cases.

The leiomyomas observed in tamoxifen-treated women do not differ histologically from those found in untreated women (Ascher et al. 2000). Liu et al. (2006) reported a case of mitotically active leiomyoma in a postmenopausal patient receiving tamoxifen. Burroughs et al. (1997) showed that tamoxifen treatment does not change the apoptotic rate of leiomyoma tissues. They suggested that growth modulation of leiomyoma by hormone modulation occurs via mechanisms independent of apoptosis. They concluded that there is a fundamental difference between the response of leiomyoma to hormone deprivation and that of tumors of the breast.

As only a few reports have studied the effect of tamoxifen on leiomyomas, the frequency of symptomatic uterine myomas and indications for surgery are unknown.

## Uterine Cervix

During long-term use, tamoxifen use has estrogenic effects in squamous epithelial cells within the cervix in postmenopausal patients (Mourits et al. 2001; Varras et al. 2003).

The association of tamoxifen with cervical polyps has been demonstrated by several studies (Varras et al. 2003). Lahti et al. (1993) found that endocervical polyps were twice as common in a tamoxifen-treated patients than in a control group. The relationship between tamoxifen use and the occurrence of cervical cancer has not been reported.

## Vagina

The vagina is lined by stratified squamous, non-keratinizing epithelium containing ER in pre- and postmenopausal women (Wiegerink et al. 1980). The epithelium is multilayered and the cells in the middle and superficial zones contain glycogen only when stimulated by estrogen. Under estrogenic stimulation, the vaginal epithelium undergoes proliferation and maturation.

Several studies have reported that tamoxifen exerts a weak estrogenic effect on the vaginal epithelium in postmenopausal patients during long-term tamoxifen use (Lahti et al. 1994; Mourits et al. 2001; Varras et al. 2003). The relationship between tamoxifen use and the occurrence of vaginal neoplasms has not been reported.

## Tumor-Like Diseases

### Endometriosis

The development of endometriosis has been reported in women receiving tamoxifen for the treatment of breast cancer (Ford et al. 1988; Cano et al. 1989; Hajjar et al. 1993; Cohen et al. 1994b; Morgan et al. 1994). A histopathologic analysis of endometriosis in a tamoxifen-treated, postmenopausal breast cancer patient showed similarity to tamoxifen-induced epithelial differentiation of the endometrium (Ismail and Maulik, 1997). The development of endometriosis has been frequently reported in postmenopausal patients taking tamoxifen (Hajjar et al. 1993; Cohen et al. 1994b; Ismail and Maulik, 1997). Several cases of endometriosis in the ovary have also been described in premenopausal women during tamoxifen administration (Ford et al. 1988; Cano et al. 1989; Morgan et al. 1994).

Endometriosis is common in premenopausal women, and its occurrence in tamoxifen-treated women in this age group may therefore be coincidental. In contrast, endometriosis is rare in postmenopausal women and the finding of endometriosis in tamoxifen-treated postmenopausal women raises the possibility of a link between tamoxifen use and endometriosis. This possibility is further supported by the unusual morphological features and behavior of endometriosis in tamoxifen treated postmenopausal women (Hajjar et al. 1993; Cohen et al. 1994a; Ismail and Maulik, 1997). One patient had pelvic endometriosis infiltrating the cervix, vagina, rectum, and sigmoid colon (Hajjar et al. 1993). Another patient had a cystic ovarian endometriotic cyst with apparent infiltration of adjacent structures (Ismail and Maulik, 1997). Another patient developed an ovarian endometrioid adenocarcinoma in an endometriotic cyst (Cohen et al. 1994b), suggesting that prolonged tamoxifen treatment may be associated with an increased risk of carcinoma arising in endometriotic foci.

As endometriosis is an estrogen-dependent disease, the mechanism by which tamoxifen acts probably that its estrogen agonistic activity stimulates the ectopic endometriotic tissue even in postmenopausal women. However, it is not clear whether tamoxifen causes de novo endometriosis or only exacerbates preexisting endometriosis.

### Adenomyosis

Several cases of adenomyosis have been reported as having developed in postmenopausal patients during tamoxifen administration (Cohen et al. 1995; Cohen et al. 1997). Cohen et al. (1997) found a higher incidence of adenomyosis (53.6%) in postmenopausal breast cancer patients treated with tamoxifen than in those not receiving the drug.

The morphological features present within adenomyosis more often in those taking tamoxifen were cystic dilatation of glands, fibrosis of the stroma, and various epithelial metaplasias (McCluggage et al. 2000). The proliferative activity within the adenomyosis was higher in the tamoxifen group. In postmenopausal tamoxifen-treated patients, ERs and PRs have been described in adenomyosis in similar concentrations as in premenopausal non-tamoxifen users (Cohen et al. 1998b).

## Conclusions

As reviewed in this paper, tamoxifen has been demonstrated to induce tumorigenesis in the female genital tract through estrogen agonism. Of the tamoxifen-associated pathologies discussed above, practitioners should be aware of the occurrence of endometrial diseases in postmenopausal women and ovarian functional cysts in premenopausal women. Tamoxifen is associated with an increased incidence of benign endometrial lesions, such as polyps and hyperplasia, as well as with a two- to three-fold increased risk of endometrial cancer in postmenopausal patients. Practitioners should be aware of the occurrence of endometrial diseases, and diagnostic procedures should be performed at the discretion of the individual gynecologist. However, there is no general consensus regarding endometrial surveillance in postmenopausal tamoxifen users. The most important recommendation by the ACOG is to thoroughly evaluate any discharge or bleeding by means of endometrial biopsy in women treated with tamoxifen (ACOG Committee Opinion, 2006) (Table 2). The risk of functional ovarian cysts is significantly high in premenopausal tamoxifen users. Cohen et al. (1994) suggested that all premenopausal breast cancer patients being treated with tamoxifen should be under close gynecological and ultrasonographic surveillance. We recommend that gynecological, cytological, and ultrasonographic examination should be performed every 4–6 months for the women receiving tamoxifen. In case of abnormal observations, further examination is necessary for the denial of malignancies.

Despite its gynecologic side effects, benefits of tamoxifen in pre- and postmenopausal breast cancer patients in controlling breast cancer or preventing its relapse are without debate. Therefore, there is a clear need to elucidate the mechanism underlying action of tamoxifen in the reproductive tract. Genital side effects of tamoxifen are an example of the complexity of its mechanism of action, with agonistic and antagonistic effects on various sites and tissues, dependent on the ambient E_2_ concentration. Frequent gynecological examination including transvaginal ultrasonography should be performed for both premenopausal and postmenopausal tamoxifen-treated patients. The multidisciplinary team including the surgeon, oncologist, and the patient’s primary care physician should be familiar with these gynecologic complications of tamoxifen therapy. Further research will enable the prediction of which groups of patients are more susceptible to develop pathologies of the genital tract.

## Figures and Tables

**Table 1.** Summary of the incidence of tamoxifen-associated gynecological malignancies.

**Site**	**Tumor type**	**Incidence/No. of reported cases**	**Status of menstruation**	**References**
Ovary	Adenocarcinoma	One case	Postmenopausal	Cohen et al. 1994b
Uterus	Endometrial cancer	0.2–0.3%	Postmenopausal	Assikis and Jordan 1995
	Sarcoma	17/100,000	Not described	Wickerham et al. 2002

**Table 2.** Recommendation on the tamoxifen use stated by ACOG Committee (ACOG Committee Opinion 2006).

Postmenopausal women taking tamoxifen should be monitored closely for symptoms of endometrial hyperplasia or cancer. Premenopausal women treated with tamoxifen have no known increased risk of uterine cancer and as such require no additional monitoring beyond routine gynecologic care. Women taking tamoxifen should be informed about the risks of endometrial proliferation, endometrial hyperplasia, endometrial cancer, and uterine sarcomas. Women should be encouraged to promptly report any abnormal vaginal symptoms, including bloody discharge, spotting, staining, or leukorrhea. Any abnormal vaginal bleeding, bloody vaginal discharge, staining, or spotting should be investigated. Emerging evidence suggests the presence of high- and low-risk groups for development of atypical hyperplasias with tamoxifen treatment in postmenopausal women based on the presence or absence of benign endometrial polyps before therapy. Thus there may be a role for pretreatment screening of postmenopausal women with transvaginal ultrasonography, and sonohysterography when needed, or office hysteroscopy before initiation of tamoxifen therapy. Unless the patient has been identified to be at high risk for endometrial cancer, routine endometrial surveillance has not been effective in increasing the early detection of endometrial cancer in women using tamoxifen. Such surveillance may lead to more invasive and costly diagnostic procedures and, therefore, is not recommended. Tamoxifen use should be limited to 5 years’ duration because a benefit beyond this time has not been documented. If atypical endometrial hyperplasia develops, appropriate gynecologic management should be instituted, and the use of tamoxifen should be reassessed. If tamoxifen therapy must be continued, hysterectomy should be considered in women with atypical endometrial hyperplasia. tamoxifen use may be reinstituted following hysterectomy for endometrial carcinoma in consultation with the physician responsible for the woman’s breast care.
